# Recurrent SARS-CoV-2 mutations at Spike D796 evade antibodies from pre-Omicron convalescent and vaccinated subjects

**DOI:** 10.1128/spectrum.03291-23

**Published:** 2024-01-08

**Authors:** Evan A. Elko, Heather L. Mead, Georgia A. Nelson, John A. Zaia, Jason T. Ladner, John A. Altin

**Affiliations:** 1 The Pathogen and Microbiome Institute, Northern Arizona University, Flagstaff, Arizona, USA; 2 The Translational Genomics Research Institute (TGen), Flagstaff, Arizona, USA; 3 Center for Gene Therapy, Department of Hematology and Hematopoietic Cell Transplantation, City of Hope National Medical Center, Duarte, California, USA; Universidade Federal do Rio de Janeiro, Rio de Janeiro, Brazil

**Keywords:** viral immunity, immune evasion, vaccination, coronavirus, assay development

## Abstract

Severe acute respiratory syndrome coronavirus 2 (SARS-CoV-2) lineages of the Omicron variant rapidly became dominant in early 2022 and frequently cause human infections despite vaccination or prior infection with other variants. In addition to antibody-evading mutations in the receptor-binding domain, Omicron features amino acid mutations elsewhere in the Spike protein; however, their effects generally remain ill defined. The Spike D796Y substitution is present in all Omicron sub-variants and occurs at the same site as a mutation (D796H) selected during viral evolution in a chronically infected patient. Here, we map antibody reactivity to a linear epitope in the Spike protein overlapping position 796. We show that antibodies binding this region arise in pre-Omicron SARS-CoV-2 convalescent and vaccinated subjects but that both D796Y and D796H abrogate their binding. These results suggest that D796Y contributes to the fitness of Omicron in hosts with pre-existing immunity to other variants of SARS-CoV-2 by evading antibodies targeting this site.

**IMPORTANCE:**

Severe acute respiratory syndrome coronavirus 2 (SARS-CoV-2) has evolved substantially through the coronavirus disease 2019 (COVID-19) pandemic: understanding the drivers and consequences of this evolution is essential for projecting the course of the pandemic and developing new countermeasures. Here, we study the immunological effects of a particular mutation present in the Spike protein of all Omicron strains and find that it prevents the efficient binding of a class of antibodies raised by pre-Omicron vaccination and infection. These findings reveal a novel consequence of a poorly understood Omicron mutation and shed light on the drivers and effects of SARS-CoV-2 evolution.

## INTRODUCTION

The Omicron variant of severe acute respiratory syndrome coronavirus 2 (SARS-CoV-2) was first identified in Botswana and South Africa in November 2021 and rapidly spread across the globe to become the predominant circulating strain. Omicron is associated with a higher reinfection rate and reduced vaccine efficacy (1, 2) and is distinguished by a striking number of mutations compared with previous variants (3, 4), which individually and collectively have the potential to alter the virus’s transmissibility, pathogenicity, and ability to escape immune responses. Of particular interest is a core set of persistent mutations shared by all circulating Omicron sub-variants: these are enriched in the Spike protein that mediates viral entry and particularly in the receptor-binding domain (RBD) within the S1 subunit, whose interaction with host ACE2 is required for infection (5). Recent studies have established that many of these Omicron RBD mutations greatly diminish the binding and/or neutralizing effects of antibodies raised by prior variants (6–8), explaining their emergence and persistence. However, Omicron also features conserved mutations outside of the RBD, including in the S2 subunit that enables membrane fusion and entry into the host cell. The consequences of these mutations are not well understood.

The persistent Omicron Spike mutation D796Y is located in the N-terminal part of the S2 subunit immediately upstream of the fusion peptide. D796Y may confer structural advantages by enhancing the interaction between the Spike-TMPRSS2 complex (9) and potentially by altering the presentation of a nearby immunogenic glycan epitope (10). Intriguingly, D796Y occurs at the same residue as a mutation (D796H) that emerged independently in 2020 in a chronically infected patient treated with remdesivir and convalescent plasma, which was shown to confer reduced sensitivity to neutralization by convalescent plasma (11). These results suggest that D796Y may have a similarly evasive effect, which is supported by a recent study showing that S2 mutations, including D796Y, can reduce the neutralization potency of S1-binding antibodies (12).

In this study, we test the hypothesis that exposure to pre-Omicron Spike proteins induces D796-binding antibodies that are evaded by both the D796H and D796Y mutations. We first use public sequence data to chart the emergence of D796 mutations throughout the pandemic and find evidence consistent with a role in conferring increased viral fitness, independently of other Omicron mutations. We then use a multiplexed and sensitive peptide-based assay to identify a public antibody epitope overlapping position D796 in cohorts with pre-Omicron infection or vaccination and to quantify the effects of the D796H and D796Y mutations on these antibody:epitope interactions.

## RESULTS

### Phylogenetic analysis of mutations at Spike codon 796 throughout the SARS-CoV-2 pandemic

**Fig 1:**
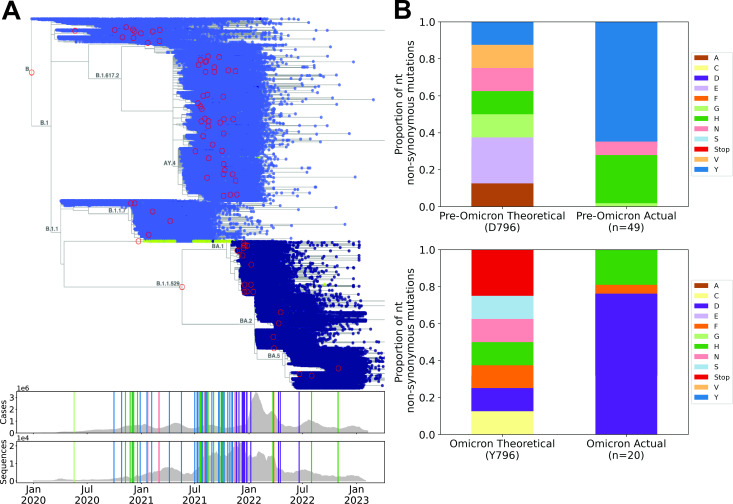
Phylogenetic analysis reveals that the D796Y and D796H substitutions occurred recurrently prior to the Omicron wave. (A) Upper: time-resolved phylogenetic tree including 6,329,365 SARS-CoV-2 sequences (Taxonium.org). The color of the points indicates the amino acid residue at position 796 of the SARS-CoV-2 Spike: light-blue, D; green, H; dark-blue, Y. Red circles highlight nodes with mutations at 796 that were found in ≥10 descendants (a threshold set to minimize the impact of technical artifacts). Branch labels indicate PANGO lineage (15). Lower: plots showing daily worldwide SARS-CoV-2 cases and total sequences per day present in the phylogenetic tree over a 2-year timescale (x-axis aligned with the phylogenetic tree in the upper panel). Vertical lines indicate the positions of mutants highlighted with red circles in the phylogenetic tree, and colors show the mutation type (matching the bar graphs in **B**). (B) The proportion of theoretically possible (left) compared with observed (right) non-synonymous amino acid substitutions resulting from single-nucleotide mutations in the codon for D796 in pre-Omicron sequences (upper) and Y796 in post-Omicron sequences (lower).

The occurrence of D796Y in all lineages of the SARS-CoV-2 Omicron variant could reflect either a positive effect on viral fitness (e.g., through antibody evasion and/or an increase in transmissibility; adaptive hypothesis) or simply physical linkage with other fitness-increasing mutations (neutral hypothesis). To begin to distinguish these possibilities, we looked for evidence of independent occurrences of D796 mutations across the SARS-CoV-2 phylogeny. We downloaded >6 million publicly available SARS-CoV-2 sequences and identified clusters containing AA mutations at position 796 and >10 descendants (Fig. 1A). The frequency of mutations identified in the phylogenetic tree was compared with the theoretical frequency of AA mutations resulting from all possible single nucleotide non-synonymous substitutions at codon 796. This analysis revealed that the D796Y and D796H substitutions have independently arisen at least 35 and 14 times throughout the pandemic, respectively, with the earliest documented instances preceding the emergence of Omicron by up to 13 months. Whereas the combined theoretical proportion of D>Y or D>H at this codon is 0.25, we observed these substitutions at a proportion >0.9 in pre-Omicron samples (*P* < 0.0001, based on 10,000 random simulations of single nucleotide non-synonymous substitution) (Fig. 1B). These findings are consistent with previous analyses (13, 14) and the hypothesis that D796Y confers a fitness advantage that is independent of other Omicron mutations.

### Infection and vaccination induce antibodies that bind a linear epitope overlapping D796

Inspection of the three-dimensional structure of the Spike trimer indicates that D796 resides on a surface-exposed loop that is theoretically available for antibody binding (Fig. S1). To test whether pre-Omicron antibody responses recognize epitopes overlapping position D796, we mapped linear epitopes at high resolution across the complete ancestral SARS-CoV-2 Spike protein by applying a highly multiplexed binding assay (“PepSeq”) to plasma from participants who were vaccinated against or convalescent from SARS-CoV-2 infection prior to the emergence of Omicron. The PepSeq platform has been previously described (16–18) and enables highly multiplexed serological analysis using programmable libraries of DNA-barcoded peptide probes that are synthesized from DNA templates in massively parallel *in vitro* reactions. The SARS-CoV-2 (SCV2) PepSeq library (17) comprises 2,500 tiled 30mer peptides of which 1,244 and 390 represent the ancestral Spike and Nucleocapsid proteins, respectively, with each peptide overlapping its neighbor by 29 amino acids. We used SCV2 to assay plasma from two cohorts sampled in 2020–2021 prior to the emergence of the Omicron variant: (i) a cohort of unvaccinated donors who were either previously infected (53 subjects) or naive (58 subjects) (“convalescent cohort”) and (ii) a cohort of 21 SARS-CoV-2 naive subjects sampled prior to and after vaccination with mRNA-1273 (“vaccinated cohort”).

**Fig 2:**
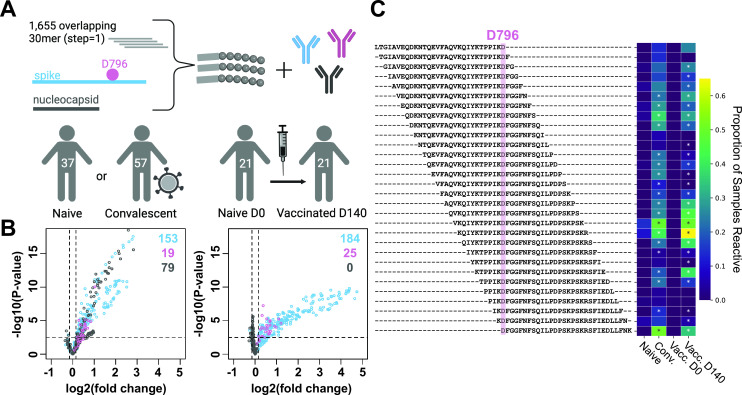
Antibody responses to D796-containing Spike peptides following natural infection with or vaccination against SARS-CoV-2. (A) A library containing 1,655 DNA-barcoded peptides tiling the complete ancestral SARS-CoV-2 Spike (S) and Nucleocapsid (N) proteins at high resolution (30mers overlapping by 29 amino acids) was synthesized using the PepSeq platform. This library was used to measure IgG reactivity at epitope resolution in naive, convalescent, and vaccinated subjects (*n* = 115 total participants). (B) Volcano plots showing the magnitude and significance of responses targeting S peptides overlapping D796 (pink), other S peptides (blue), and N peptides (gray), in comparisons of naive versus convalescent participants (left) and naive versus vaccinated timepoints (right). Plots show 451 peptides that met our threshold of having a Z score > 20 in at least one sample. Each point indicates an individual peptide. Dashed lines indicate thresholds at log2(fold changes) of ±0.15 and −log10(*P*-value) of 2.5. Numbers in the top-right corner indicate the number of S peptides overlapping D796 (pink), other S peptides (blue), and N peptides (gray) that meet the indicated fold change and *P*-value thresholds. (C) Heatmap showing the proportion of participants who were reactive to each peptide (at a Z score threshold ≥ 6) in the indicated cohort across all 30 peptides that contained D796 (y-axis). Asterisks indicate peptides whose responses exceed the cohort-wide thresholds shown in B. Exact proportions can be found in Table S3.

To identify epitopes recognized in response to infection or vaccination, we compared reactivity between infected and naive subjects in the convalescent cohort and matched pre- and post-vaccination samples in the vaccinated cohort (Fig. 2A). This analysis revealed robust antibody responses to infection and vaccination: we detected significantly increased reactivity in 172/79 and 209/0 Spike/Nucleocapsid peptides in the convalescent and vaccinated cohorts, respectively (hereafter termed “responding peptides”). The restriction of Nucleocapsid-responding peptides to the convalescent cohort is expected, consistent with the lack of this protein sequence in the mRNA-1273 vaccine. Importantly, we observed strong responses to peptides overlapping position D796 in both cohorts: among the 30 assayed peptides tiled across this position, 19 and 25 were significantly elevated in the convalescent and vaccinated cohorts, respectively (Fig. 2B and C), with up to 2.3-fold increases in enrichment Z scores. The response frequencies among peptides overlapping D796 (0.63 and 0.83 in convalescent and vaccinated subjects, respectively) are substantially higher than the corresponding rates among the overall set of 1,244 Spike peptides (0.14 and 0.17).

The positions of responding peptides within the Spike protein were highly similar between the convalescent and vaccinated cohorts (Fig. S2) and closely match previously described epitope regions, including the fusion peptide and stem helix regions against which broadly neutralizing antibodies have been described (19–21). Consistent with previous studies using peptide-based serology (16, 17, 22, 23), we observed little reactivity to probes designed from the RBD or N-terminal domain regions. Antibody epitopes in these regions tend to recognize conformational epitopes that are not well represented by peptide antigens (24, 25).

### Quantifying the impact of D796 mutations on antibody binding

**Fig 3:**
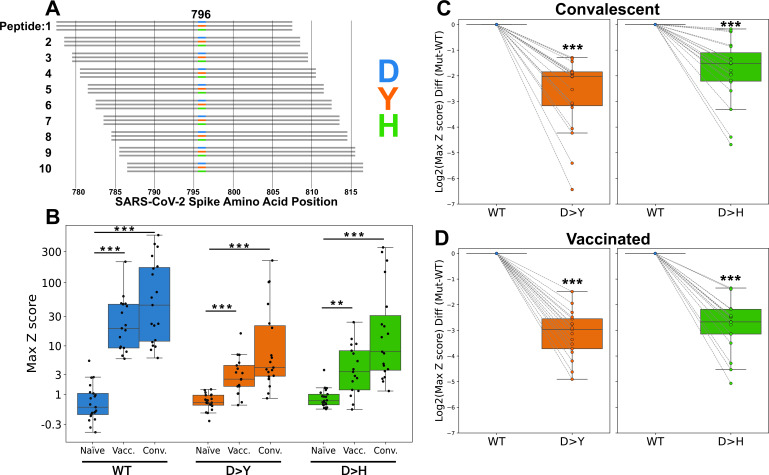
D796Y and D796H mutations abrogate the binding of infection- and vaccine-induced antibodies to Spike peptides. (A) A PepSeq library was generated to compare three different residues at Spike position 796—D (WT), Y, and H—each replicated in 10 tiled overlapping peptides (total 30 peptides containing position 796). (B) Reactivity for naive participants compared with convalescent and vaccinated day ~140 participants for WT (D796, blue) and mutant (Y796, orange; H796, green) peptides. (C and D) Reactivity differences between mutant (Y796, H796) and WT (D796) peptides in convalescent participants (upper, *n* = 19) and vaccinated participants at day ~140 (lower, *n* = 17). Y-axes in B through D show maximum reactivity among the 10 overlapping peptides containing the indicated amino acid at position 796. For C and D, mutant reactivity was normalized in each case to the WT signal from the same sample. Data for all 10 individual peptides aggregated in panels B through D can be found in Fig. S3 and S4. **P < 0.01 and ***P < 0.001 by Mann-Whitney U test (**B**) or Wilcoxon signed-rank test (C and D).

Having identified antibody reactivity in pre-Omicron immune subjects to a region of the Spike protein that overlaps D796, we next sought to test the hypothesis that the D796Y and D796H mutants evade these responses. We quantified antibody binding to three versions of this position—wild-type (WT) (D796), D796Y, and D796H—in the pre-Omicron immune cohorts described above. To accomplish this, we generated a new PepSeq library in which we represented each of the three versions of position 796 in the form of 10 overlapping individual 30mer peptides (Fig. 3A; minimum overlap = 21 amino acids). We used this library to study the binding specificity of antibodies in subjects selected according to their reactivity to the region containing position 796 (19 convalescent subjects at days 27–314 after testing positive for SARS-CoV-2 and 17 vaccinated subjects at day ~140 post-vaccination) (Fig. 3; Fig. S3 and S4). As expected, signals from each of the individual overlapping peptides, for each genotype, were broadly consistent; however, we did observe some peptide-to-peptide variability in both absolute and relative levels of reactivity that may be due to differences in peptide abundance, secondary structure, and/or coverage of antibody-binding residues (Fig. S3 and S4). To account for peptide-to-peptide variability, we aggregated these signals for each participant by taking the maximum Z score across each set of 10 overlapping peptides.

This aggregated analysis revealed a significant increase in antibody reactivity to all three genotypes (D796, Y796, and H796) in both the vaccinated day ~140 and convalescent groups compared with naive subjects (Fig. 3B). However, we also observed a marked and consistent reduction in antibody reactivity to peptides containing either the D796Y or D796H substitutions in both cohorts, compared with the unmutated versions (Fig. 3C and D). Both of these effects were broadly consistent across each of the 10 tiled peptides for each mutation (Fig. S3 and S4). We conclude that the mutation of D to either Y or H evades a large portion (but not all) of the antibody response against a linear epitope overlapping position 796 induced by exposure to ancestral Spike proteins.

## DISCUSSION

In this study, we map antibody binding to an epitope that overlaps residue D796 of SARS-CoV-2 Spike: a residue that has mutated to Y and H recurrently throughout the pandemic, including in a well-characterized chronically infected patient (D796H), and is now fixed in all Omicron subvariants (D796Y). By studying convalescent and vaccinated cohorts, we show that this epitope is frequently targeted by the polyclonal antibody response to ancestral Spike proteins but that antibody binding is significantly abrogated by both the D796H and D796Y mutations. Together, these results reveal a novel immunological consequence of these important Spike mutations and raise the possibility that immune pressure mediated by antibodies binding to this epitope in the Spike S2 subunit has helped to shape the evolution of SARS-CoV-2.

Although we observe antibodies binding the D796-containing epitope in a high fraction of subjects (>70% of convalescent/vaccinated subjects; Fig. 2C; Fig. S2B), their functional consequences remain to be determined. Previous work indicated that D796H reduces sensitivity to neutralization by convalescent plasma; however, this mutation alone also causes an opposing and marked intrinsic infectivity defect that is unrelated to antibodies (11). In light of our observation that D796Y and D796H both evade antibodies to a linear epitope containing position 796 to similar degrees (Fig. 3C and D), we hypothesize that D796Y may likewise confer a partial neutralization-evading effect. Mechanistically, the proximity of this epitope to the S2’ cleavage site raises the possibility that such antibodies could neutralize SARS-CoV-2 by inhibiting S2’ cleavage by TMPRSS2, an event that is necessary for host cell infection (26). Alternatively, or additionally, these antibodies may act against SARS-CoV-2 through neutralization-independent mechanisms.

In addition to the class of antibodies defined here, D796 mutations may evade antibodies binding Spike at distal sites through complex conformational changes, as indicated by the recent observation that D796Y decreases sensitivity to neutralizing antibodies binding the S1 subunit (12). This reveals an intriguing potential for multi-epitope evasion by a single mutation, although the relative contributions of these effects to overall immune evasion remain to be determined. Future studies will also be important to study how immune responses to these regions evolve following exposure to Spike proteins containing D796Y (through infection and/or vaccination).

By identifying a population of antibodies whose binding depends on D796, our results suggest immune pressure as an important driver of the emergence of D796 mutations, which would be consistent with its role in shaping the evolution of SARS-CoV-2 more broadly. A more complete understanding of the mutational pathways by which SARS-CoV-2 has evaded, and continues to evade, immune responses may enable better prediction of its future evolution, as well as the identification of evasion-resistant vulnerabilities that can be exploited in the search for next-generation therapies and vaccines.

## MATERIALS AND METHODS

### Phylogenetic analysis

The phylogenetic tree of 6,329,365 publicly available SARS-CoV-2 sequences from GenBank (i.e., the INSDC databases), from the China National Center for Bioinformation, and from COG-UK, along with estimated tip dates, was downloaded from https://taxonium.org/?backend=https://api.cov2tree.org on 8 February 2023. If no date was supplied for a given sequenced genome, tip dates were estimated using Chronumental (27). These tip dates were then used to calculate the number of sequences per day in the data set. The number of global SARS-CoV-2 cases per day was downloaded from https://ourworldindata.org/covid-cases on 9 February 2023. Taxonium (28) was used to visualize the phylogenetic tree and identify branches with mutations at D796 that had greater than 10 descendants. Nodes meeting this criteria were then used to calculate the proportion of single nucleotide non-synonymous amino acid mutations pre- and post-omicron becoming the dominant global variant. The theoretical distribution of single nucleotide non-synonymous amino acid mutations was calculated by considering all possible amino acid outcomes of single nucleotide mutations at the D796 codon. A simulation approach was utilized to determine the likelihood that the observed AA mutation frequency was caused by random chance based on the resulting theoretical AA distribution of single nucleotide non-synonymous substitutions. Ten thousand iterations were run using the theoretical distributions pre- and post-Omicron. For each iteration, the number of observed mutations pre- and post-Omicron (*n* = 49 and *n* = 20, respectively) was chosen from the theoretical distribution and the proportion of D > Y/H mutations was calculated and compared to the actual observed proportion of D>Y/H mutations.

### Study subjects

Under an IRB-approved study (IRB#20204, NCT04497779), unvaccinated coronavirus disease 2019 (COVID-19) convalescent individuals were recruited from participating clinical sites. Participants all experienced mild COVID-19 symptoms and donated blood within 7–142 days of their initial PCR-based diagnosis. Age, sex, whether they were positive or negative for SARS-CoV-2 spike IgG antibody, and sample collection dates are listed in Table S1. Pre-pandemic naive control serum samples (*n* = 24) were collected by Creative Testing Solutions (Phoenix, AZ) during January 2015 from multiple locations in California (Table S1). The use of all pre-pandemic samples was reviewed by the TGen and NAU Research Compliance offices and determined not to be human subject research.

The vaccinated cohort studied here has been previously described (16, 17). Under an IRB-approved study (WIRB#1299650), 21 healthy participants were recruited from a local research institution and the surrounding community. Subjects donated blood prior to their first dose (baseline) of the Moderna COVID-19 vaccine (mRNA-1273) and then approximately 140 days from baseline, after receiving both recommended vaccine doses (“day 140”). The characteristics and exact collection timepoints for each donor are listed in Table S2.

### PepSeq libraries

The SCV2 PepSeq library contained 2,500 unique 30mer peptides and was designed to provide high-resolution coverage of the SARS-CoV-2 Spike and Nucleocapsid proteins, as previously described (17). Briefly, this library included all unique 30-amino acid peptides contained in consensus versions of the SARS-CoV-2 Spike and Nucleocapsid proteins generated early in the COVID-19 pandemic. This included 1,244 Spike and 390 Nucleocapsid peptides, each of which was represented by three different nucleotide encodings.

The 796 mutant library is a derivative of our human virome version 2 PepSeq library, which has been described in detail previously (16). It contains 15,000 unique 30mer peptides including a set of 30 peptides designed to test the impact of the mutations at position 796 of the SARS-CoV-2 Spike: each of three residues (D, Y, and H) was represented in 10 overlapping peptides (tiled with a step of one amino acid, as shown in Fig. 3A).

### PepSeq library synthesis and antibody-binding assay

The SCV2 and 796 mutant library designs were encoded as libraries of 7,500 or 15,000 DNA oligonucleotides, respectively. These oligonucleotides were used to synthesize a corresponding “PepSeq” library of DNA-barcoded peptides for multiplexed analysis of antibody reactivity profiles, as previously described (29). Briefly, the oligonucleotide library was PCR amplified and then used to generate mRNA in an *in vitro* transcription reaction. The products were ligated to hairpin oligonucleotide adapters bearing a puromycin molecule tethered by a PEG spacer and used as a template in an *in vitro* translation reaction. Finally, a reverse transcription reaction, primed by the adaptor hairpin, was used to generate cDNA, and the original mRNA was removed using RNAse. To perform serological assays, the resulting DNA-barcoded peptide library was added to diluted plasma and incubated overnight. The binding reaction was applied to pre-washed protein G-bearing beads, washed, eluted, and indexed using barcoded DNA oligos. Following PCR cleanup, products were pooled, quantified, and sequenced using an Illumina NextSeq instrument resulting in the generation of between 47,383 and 3,917,807 reads per sample.

### PepSeq data analysis

PepSeq sequencing data were processed and analyzed as previously described (17) using PepSIRF v1.6.0 (30, 31), as well as custom scripts (https://github.com/LadnerLab/PepSIRF/tree/master/extensions). First, the reads were demultiplexed and assigned to peptides using the PepSIRF *demux* module, allowing for one mismatch in each index sequence and two mismatches in the variable DNA tag region. The PepSIRF *norm* module was then used to normalize counts to reads per million (RPM). RPM-normalized reads from seven buffer-only negative control assays were used to create bins for Z score calculation using the PepSIRF *bin* module. To normalize for different starting peptide abundances within each bin, reads were further normalized by subtracting the average RPM from the buffer-only controls (--diff option in *norm* module). Z scores were calculated using the PepSIRF *zscore* module using the 70% (SCV2) or 60% (796 mutant library) highest-density interval within each bin. The peptide Z scores were compared between naive/convalescent and naive/vaccinated cohorts using Student’s *t*-test, and peptides were considered significantly responsive if they had a log2(fold change) ≥ 0.15 and a −log10(*P* value) ≥ 2.5. The proportion of samples in each cohort that contained an enriched peptide (Z scores ≥ 6) was calculated for all peptides overlapping the SARS-CoV-2 spike protein position 796.

## Data Availability

The raw peptide counts and Z score data from this study have been deposited in the Open Science Framework (https://osf.io/h6nty/). All custom code is available via GitHub (https://github.com/LadnerLab). Any additional information required to reanalyze the data reported in this paper is available from the lead contact upon request.

## SUPPLEMENTAL MATERIAL

Figure S1 spectrum.03291-23-s0001.tiff 3D structure of Omicron Spike protein in the pre-fusion conformation highlighting Y796.

Figure S2 spectrum.03291-23-s0002.tif Spike and nucleocapsid protein-wide linear epitope reactivity in convalescent and vaccinated subjects detected using PepSeq.

Figure S3 spectrum.03291-23-s0003.tif Antibody reactivity to peptides tiled across Spike position 796 in vaccinated and convalescent subjects.

Figure S4 spectrum.03291-23-s0004.tif Effect of D>Y and D>H mutations on convalescent and vaccinated reactivity to individual tiled peptides covering position 796.

Supplemental Tables spectrum.03291-23-s0005.docx Tables S1, S2, and S3.

ASM does not own the copyrights to Supplemental Material that may be linked to, or accessed through, an article. The authors have granted ASM a non-exclusive, world-wide license to publish the Supplemental Material files. Please contact the corresponding author directly for reuse.
