# Supplementary figures and images for: Recurrent SARS-CoV-2 mutations at Spike D796 evade antibodies from pre-Omicron convalescent and vaccinated subjects
Source: Microbiol Spectr. 2024 Jan 8;12(2):e03291-23. doi: 10.1128/spectrum.03291-23 (PMC10871546; doi:10.1128/spectrum.03291-23)

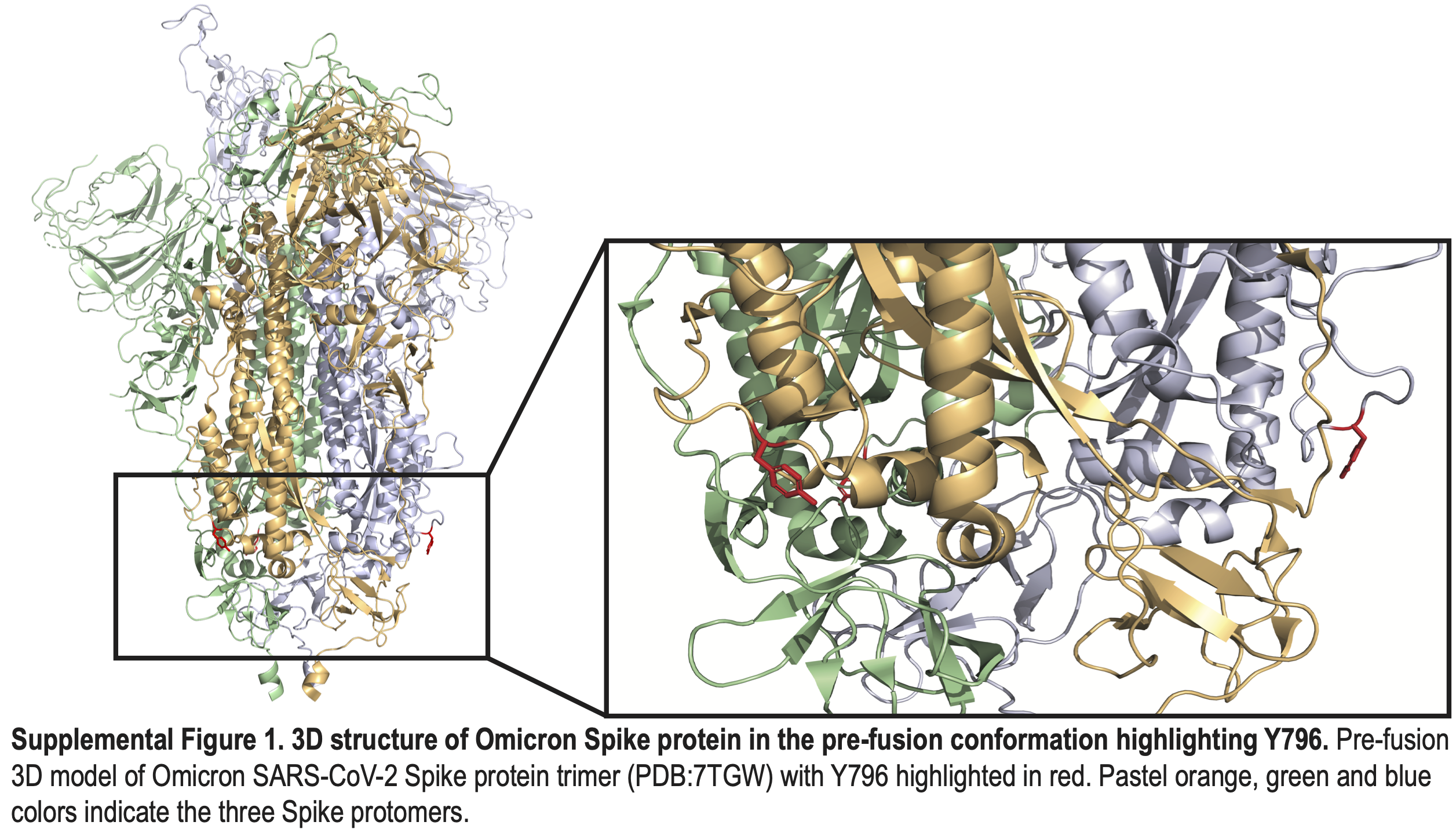

Supplement: Figure S1 — 3D structure of Omicron Spike protein in the pre-fusion conformation highlighting Y796. [file spectrum.03291-23-s0001.tiff]

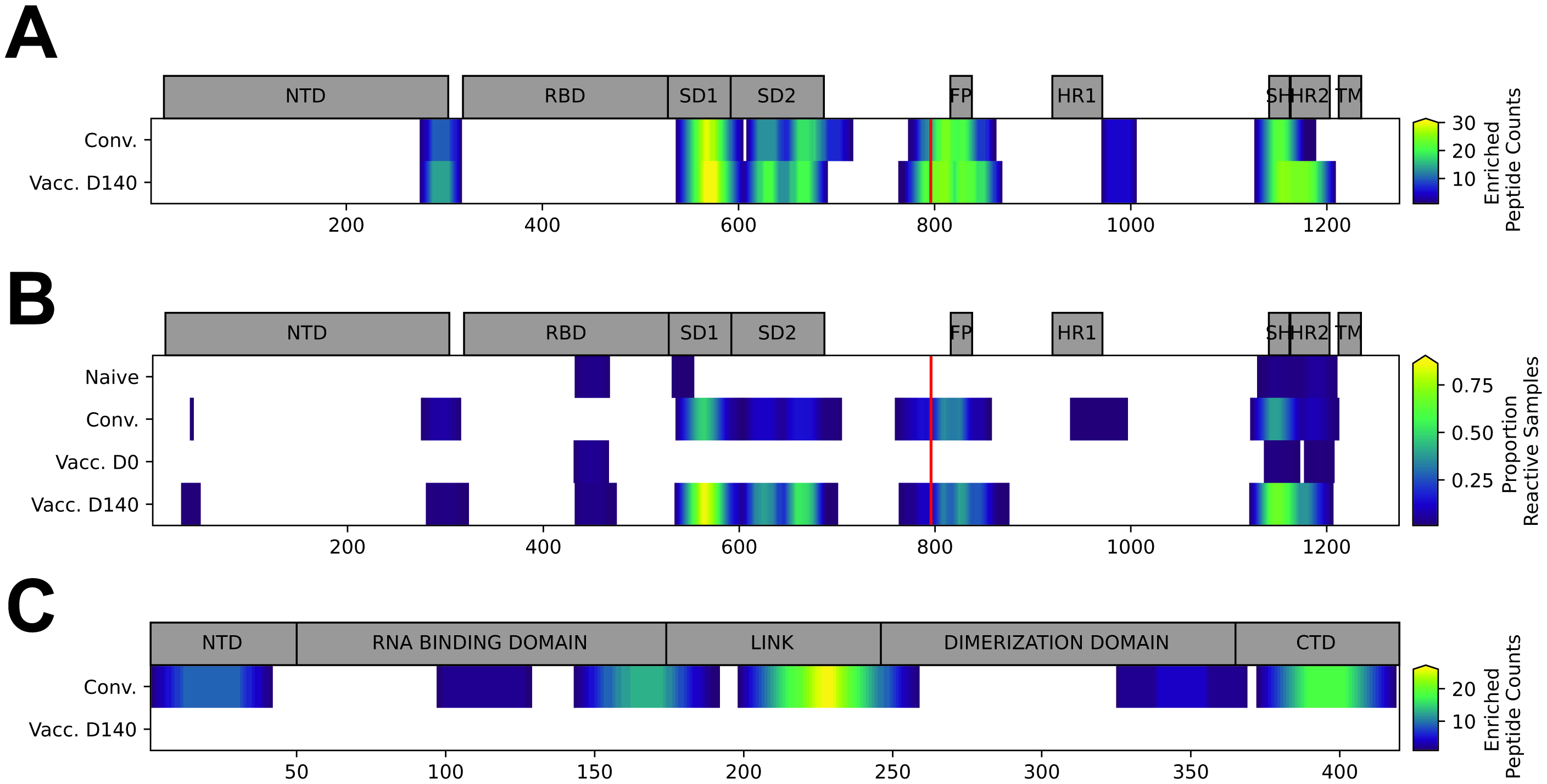

Supplement: Figure S2 — Spike and nucleocapsid protein-wide linear epitope reactivity in convalescent and vaccinated subjects detected using PepSeq. [file spectrum.03291-23-s0002.tif]

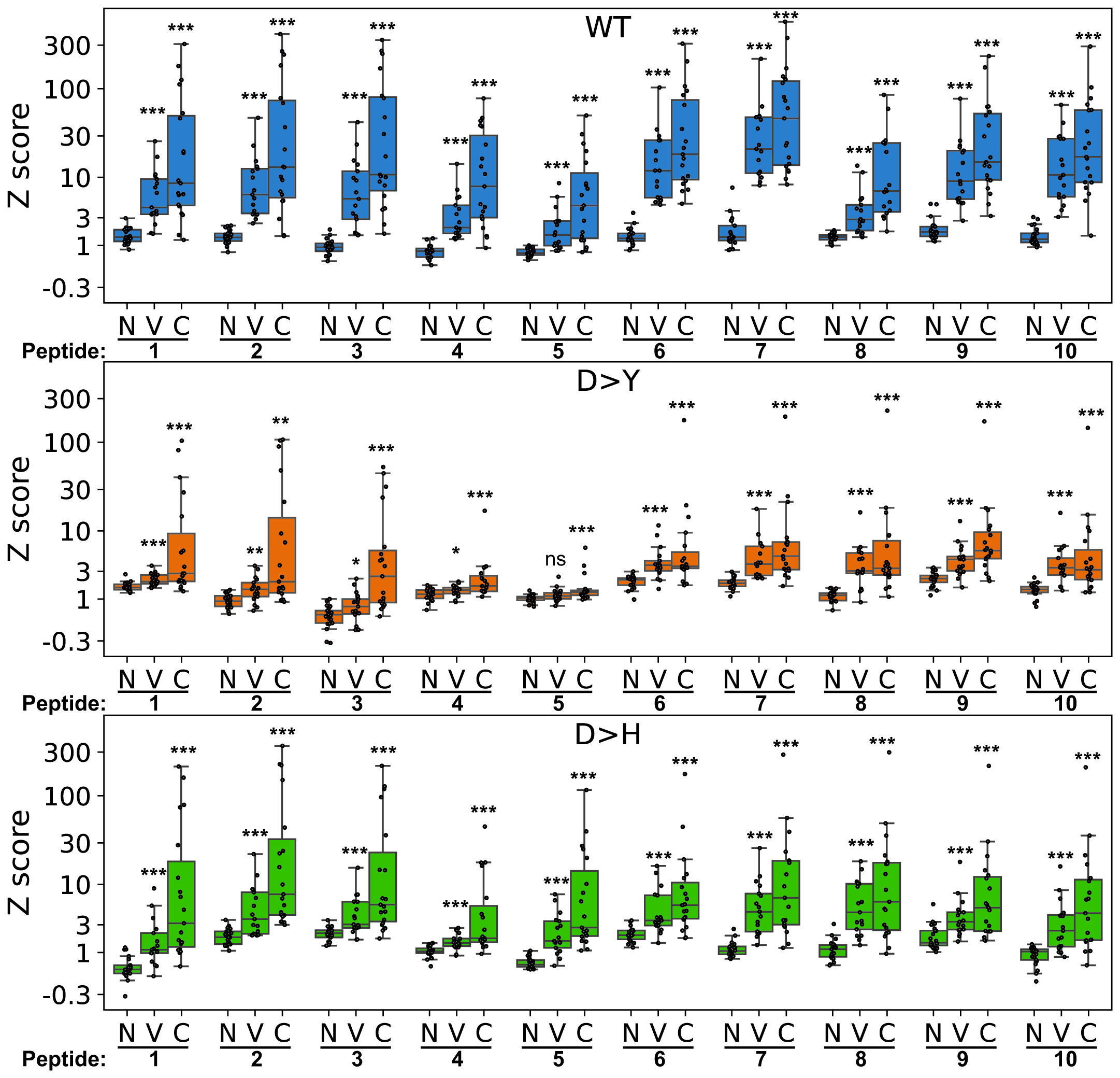

Supplement: Figure S3 — Antibody reactivity to peptides tiled across Spike position 796 in vaccinated and convalescent subjects. [file spectrum.03291-23-s0003.tif]

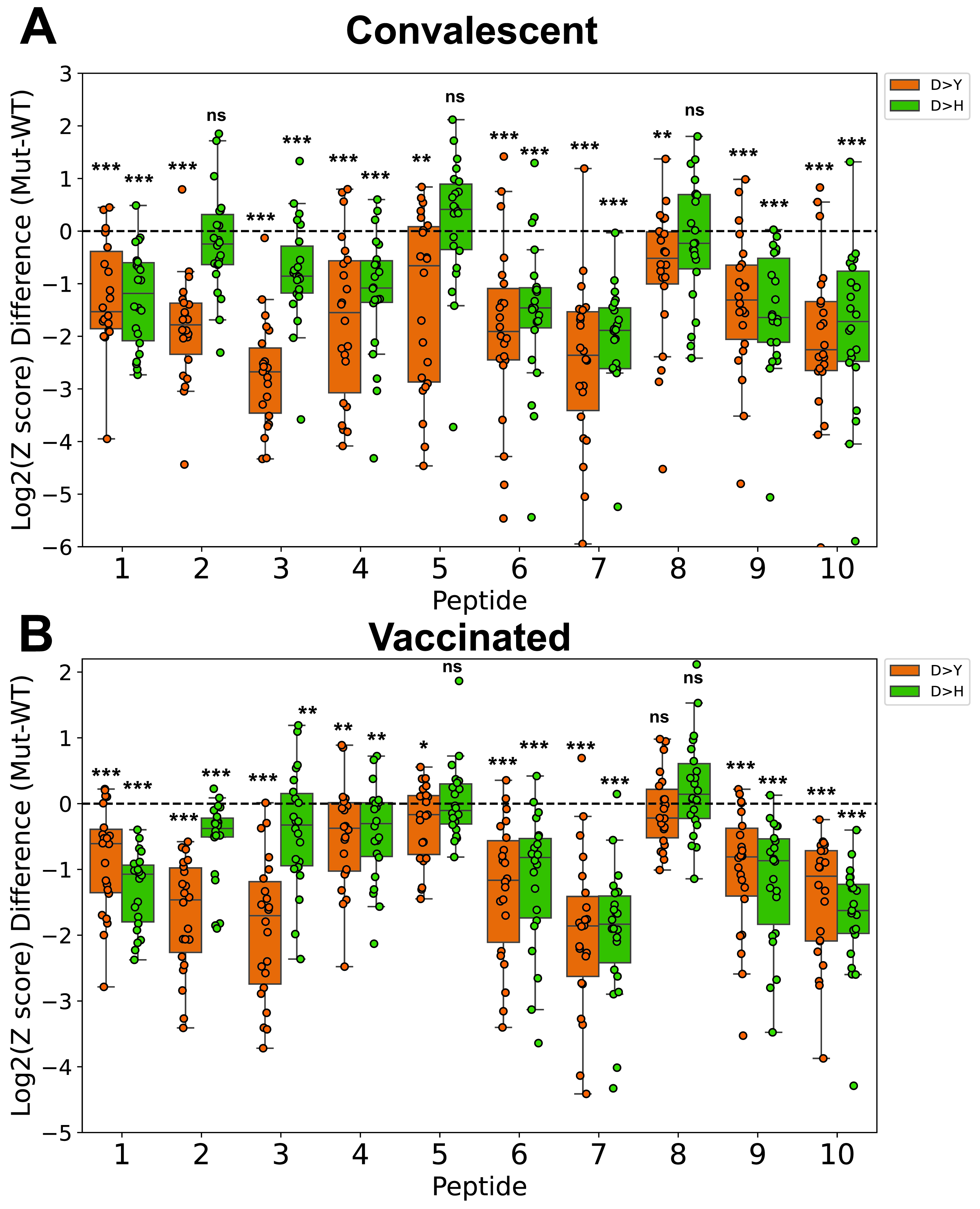

Supplement: Figure S4 — Effect of D>Y and D>H mutations on convalescent and vaccinated reactivity to individual tiled peptides covering position 796. [file spectrum.03291-23-s0004.tif]
