# Supplementary material for: Recurrent SARS-CoV-2 mutations at Spike D796 evade antibodies from pre-Omicron convalescent and vaccinated subjects
Source: Microbiol Spectr. 2024 Jan 8;12(2):e03291-23. doi: 10.1128/spectrum.03291-23 (PMC10871546; doi:10.1128/spectrum.03291-23)
Supplement: Supplemental Tables — Tables S1, S2, and S3. [file spectrum.03291-23-s0005.docx]

**Supplemental Table 1 Convalescent and Naive Participant Biodata**

| Sample ID | Age | Sex | Collection date | Category | PepSeq Library |
| --- | --- | --- | --- | --- | --- |
| BB001 | 19 | M | 1/6/2015 | naive | SCV2 |
| BB008 | 37 | M | 1/7/2015 | naive | SCV2 |
| BB009 | 20 | F | 1/5/2015 | naive | SCV2 |
| BB010 | 22 | F | 1/5/2015 | naive | SCV2 |
| BB012 | 23 | M | 1/7/2015 | naive | SCV2 |
| BB013 | 61 | M | 1/6/2015 | naive | SCV2 |
| BB015 | 56 | M | 1/7/2015 | naive | SCV2 |
| BB017 | 56 | M | 1/9/2015 | naive | SCV2 |
| BB018 | 53 | F | 1/9/2015 | naive | SCV2 |
| BB019 | 45 | M | 1/9/2015 | naive | SCV2 |
| BB401 | 40 | M | 5/5/2015 | naive | SCV2 |
| BB402 | 32 | M | 5/5/2015 | naive | SCV2 |
| BB403 | 68 | F | 5/5/2015 | naive | SCV2 |
| BB404 | 38 | M | 5/5/2015 | naive | SCV2 |
| BB405 | 52 | M | 5/5/2015 | naive | SCV2 |
| BB406 | 32 | F | 5/5/2015 | naive | SCV2 |
| BB407 | 64 | F | 5/5/2015 | naive | SCV2 |
| BB408 | 64 | F | 5/5/2015 | naive | SCV2 |
| BB409 | 42 | F | 5/5/2015 | naive | SCV2 |
| BB410 | 20 | M | 5/5/2015 | naive | SCV2 |
| BB411 | 57 | M | 5/5/2015 | naive | SCV2 |
| BB412 | 52 | M | 5/5/2015 | naive | SCV2 |
| BB413 | 65 | F | 5/5/2015 | naive | SCV2 |
| BB414 | 34 | F | 5/5/2015 | naive | SCV2 |
| CoH-P-4 | 38 | M | 8/6/20 | Convalescent | SCV2 |
| CoH-P-5 | 47 | M | 8/4/20 | Convalescent | SCV2 |
| CoH-P-6 | 66 | M | 10/7/20 | Convalescent | SCV2 |
| CoH-P-7 | 99 | M | 7/27/20 | Convalescent | SCV2 & 796 mutant |
| CoH-P-8 | 54 | F | 7/22/20 | Naive | SCV2 |
| CoH-P-9 | 35 | M | 8/21/20 | Convalescent | SCV2 |
| CoH-P-10 | 47 | F | 9/2/20 | Naive | SCV2 & 796 mutant |
| CoH-P-11 | 57 | M | 7/21/20 | Naive | SCV2 |
| CoH-P-12 | 64 | M | 10/6/20 | Convalescent | SCV2 |
| CoH-P-13 | 32 | M | 8/10/20 | Convalescent | SCV2 |
| CoH-P-14 | 35 | F | 12/11/20 | Convalescent | SCV2 |
| CoH-P-15 | 71 | M | 6/1/21 | Convalescent | SCV2 |
| CoH-P-16 | NC | M | 10/20/21 | Convalescent | SCV2 & 796 mutant |
| CoH-P-17 | NC | F | 10/20/21 | Convalescent | SCV2 & 796 mutant |
| CoH-P-18 | 50 | F | 11/19/20 | Convalescent | SCV2 |
| CoH-P-19 | NC | M | 11/12/21 | Convalescent | SCV2 |
| CoH-P-20 | 55 | F | 9/16/20 | Naive | SCV2 |
| CoH-P-21 | 58 | F | 7/8/21 | Convalescent | SCV2 & 796 mutant |
| CoH-P-22 | 52 | F | 6/1/21 | Convalescent | SCV2 & 796 mutant |
| CoH-P-23 | 52 | M | 1/11/22 | Convalescent | SCV2 |
| CoH-P-24 | 53 | F | 1/26/21 | Naive | SCV2 |
| CoH-P-25 | NC | F | 12/2/21 | Convalescent | SCV2 |
| CoH-P-26 | 27 | M | 2/12/21 | Convalescent | SCV2 & 796 mutant |
| CoH-P-27 | 70 | F | 6/1/21 | Convalescent | SCV2 & 796 mutant |
| CoH-P-28 | 70 | F | 11/3/20 | Convalescent | 796 mutant |
| CoH-P-29 | 51 | F | 4/13/21 | Convalescent | SCV2 |
| CoH-P-30 | 31 | M | 10/5/20 | Convalescent | SCV2 & 796 mutant |
| CoH-P-31 | 36 | F | 12/8/20 | Convalescent | SCV2 |
| CoH-P-32 | 63 | F | 6/14/21 | Convalescent | SCV2 |
| CoH-P-33 | 68 | F | 3/17/21 | Convalescent | SCV2 |
| CoH-P-34 | 33 | M | 6/8/21 | Convalescent | SCV2 & 796 mutant |
| CoH-P-35 | 59 | F | 10/13/21 | Convalescent | SCV2 |
| CoH-P-36 | 56 | M | 5/24/21 | Convalescent | SCV2 |
| CoH-P-37 | 38 | M | 2/22/21 | Naive | SCV2 |
| CoH-P-38 | 51 | F | 9/1/21 | Convalescent | SCV2 & 796 mutant |
| CoH-P-39 | 52 | M | 12/18/20 | Convalescent | SCV2 & 796 mutant |
| CoH-P-40 | 70 | M | 6/14/21 | Convalescent | SCV2 |
| CoH-P-41 | 73 | M | 5/25/21 | Convalescent | SCV2 |
| CoH-P-42 | 34 | F | 10/11/21 | Naive | SCV2 |
| CoH-P-43 | 29 | F | 12/11/20 | Convalescent | SCV2 |
| CoH-P-44 | 83 | M | 7/7/21 | Convalescent | SCV2 |
| CoH-P-45 | 53 | F | 5/3/21 | Convalescent | SCV2 |
| CoH-P-46 | 18 | F | 10/2/20 | Convalescent | SCV2 & 796 mutant |
| CoH-P-47 | 56 | F | 11/5/20 | Convalescent | 796 mutant |
| CoH-P-48 | NC | F | 8/25/21 | Convalescent | SCV2 |
| CoH-P-49 | NC | F | 11/16/21 | Convalescent | SCV2 |
| CoH-P-50 | 61 | M | 5/20/21 | Convalescent | SCV2 |
| CoH-P-51 | 43 | M | 5/27/21 | Convalescent | SCV2 |
| CoH-P-52 | 36 | M | 7/29/20 | Naive | SCV2 |
| CoH-P-53 | 70 | F | 7/21/20 | Naive | SCV2 |
| CoH-P-54 | 48 | F | 10/8/20 | Convalescent | SCV2 & 796 mutant |
| CoH-P-55 | 41 | F | 7/16/20 | Naive | SCV2 |
| CoH-P-56 | 26 | M | 8/20/20 | Convalescent | SCV2 & 796 mutant |
| CoH-P-57 | 48 | M | 8/28/20 | Convalescent | SCV2 & 796 mutant |
| CoH-P-58 | 57 | M | 8/10/20 | Convalescent | SCV2 |
| CoH-P-59 | 31 | M | 10/1/20 | Naive | SCV2 |
| CoH-P-60 | 42 | F | 8/10/20 | Convalescent | SCV2 & 796 mutant |
| CoH-P-61 | 49 | F | 9/22/20 | Naive | SCV2 |
| CoH-P-62 | 41 | M | 9/30/20 | Convalescent | SCV2 |
| CoH-P-63 | 23 | F | 9/29/20 | Convalescent | SCV2 |
| CoH-P-64 | 33 | M | 8/24/20 | Convalescent | SCV2 |
| CoH-P-65 | 45 | M | 8/28/20 | Convalescent | SCV2 |
| CoH-P-66 | 61 | M | 8/5/20 | Convalescent | SCV2 |
| CoH-P-67 | 53 | F | 9/30/20 | Convalescent | 796 mutant |
| CoH-P-68 | 65 | M | 10/8/20 | Convalescent | SCV2 |
| CoH-P-69 | 41 | F | 11/4/20 | Convalescent | 796 mutant |
| CoH-P-70 | 39 | M | 8/12/20 | Naive | SCV2 |
| CoH-P-71 | 71 | F | 8/12/20 | Naive | SCV2 |
| CoH-P-72 | 51 | F | 10/6/20 | Convalescent | SCV2 & 796 mutant |
| CoH-P-73 | 32 | F | 8/4/20 | Convalescent | SCV2 & 796 mutant |
| CoH-P-74 | 29 | F | 7/17/20 | Convalescent | SCV2 |

NC= Not Collected

**Supplemental Table 2 Vaccinated Participant Biodata**

| Sample ID | Period between doses (days) | Collection timepoints  (days from first vaccine dose) | | PepSeq Library |
| --- | --- | --- | --- | --- |
|  |  | ~Day 0 | ~Day 140 |  |
| TGen 552 | 28 | 0 | 140 | SCV2 & 796 mutant |
| TGen 827 | 28 | 0 | 138 | SCV2 & 796 mutant |
| TGen 296 | 28 | 0 | 133 | SCV2 & 796 mutant |
| TGen 625 | 28 | 0 | 135 | SCV2 & 796 mutant |
| TGen 030 | 28 | 0 | 138 | SCV2 & 796 mutant |
| TGen 636 | 32 | 1 | 138 | SCV2 & 796 mutant |
| TGen 669 | 28 | 0 | 139 | SCV2 & 796 mutant |
| TGen 846 | 28 | 0 | 140 | SCV2 & 796 mutant |
| TGen 215 | 32 | 0 | 140 | SCV2 & 796 mutant |
| TGen 263 | 29 | 0 | 135 | SCV2 & 796 mutant |
| TGen 049 | 28 | 0 | 140 | SCV2 & 796 mutant |
| TGen 406 | 27 | 1 | 137 | SCV2 & 796 mutant |
| TGen 385 | 32 | 0 | 138 | SCV2 & 796 mutant |
| TGen 656 | 28 | 0 | 138 | SCV2 & 796 mutant |
| TGen 137 | 28 | 0 | 133 | SCV2 & 796 mutant |
| TGen 083 | 28 | 2 | 134 | SCV2 & 796 mutant |
| TGen 472 | 28 | 0 | 133 | SCV2 & 796 mutant |
| TGen 007 | 28 | 1 | NC | SCV2 & 796 mutant |
| TGen 534 | 28 | 8 | 141 | SCV2 & 796 mutant |
| TGen 954 | 28 | 8 | 141 | SCV2 & 796 mutant |

NC= Not Collected, *Prior to first vaccine

All timepoints are expressed in days relative to the day on which the first vaccine dose occurred.

**Supplemental Table 3 Proportion of participants reactive to each peptide**

| Peptide | Naïve | Convalescent | Vaccinated Day 0 | Vaccinated Day 140 |
| --- | --- | --- | --- | --- |
| LTGIAVEQDKNTQEVFAQVKQIYKTPPIK**D** | 0.00 | 0.13 | 0.00 | 0.25 |
| TGIAVEQDKNTQEVFAQVKQIYKTPPIK**D**F | 0.00 | 0.13 | 0.00 | 0.00 |
| GIAVEQDKNTQEVFAQVKQIYKTPPIK**D**FG | 0.02 | 0.21 | 0.00 | 0.25 |
| IAVEQDKNTQEVFAQVKQIYKTPPIK**D**FGG | 0.00 | 0.17 | 0.00 | 0.15 |
| AVEQDKNTQEVFAQVKQIYKTPPIK**D**FGGF | 0.03 | 0.25 | 0.00 | 0.20 |
| VEQDKNTQEVFAQVKQIYKTPPIK**D**FGGFN | 0.00 | 0.23 | 0.00 | 0.30 |
| EQDKNTQEVFAQVKQIYKTPPIK**D**FGGFNF | 0.03 | 0.28 | 0.00 | 0.20 |
| QDKNTQEVFAQVKQIYKTPPIK**D**FGGFNFS | 0.03 | 0.38 | 0.00 | 0.30 |
| DKNTQEVFAQVKQIYKTPPIK**D**FGGFNFSQ | 0.00 | 0.30 | 0.00 | 0.20 |
| KNTQEVFAQVKQIYKTPPIK**D**FGGFNFSQI | 0.00 | 0.06 | 0.00 | 0.00 |
| NTQEVFAQVKQIYKTPPIK**D**FGGFNFSQIL | 0.00 | 0.04 | 0.00 | 0.00 |
| TQEVFAQVKQIYKTPPIK**D**FGGFNFSQILP | 0.00 | 0.23 | 0.00 | 0.05 |
| QEVFAQVKQIYKTPPIK**D**FGGFNFSQILPD | 0.02 | 0.25 | 0.00 | 0.15 |
| EVFAQVKQIYKTPPIK**D**FGGFNFSQILPDP | 0.00 | 0.26 | 0.00 | 0.00 |
| VFAQVKQIYKTPPIK**D**FGGFNFSQILPDPS | 0.00 | 0.11 | 0.00 | 0.00 |
| FAQVKQIYKTPPIK**D**FGGFNFSQILPDPSK | 0.00 | 0.17 | 0.00 | 0.15 |
| AQVKQIYKTPPIK**D**FGGFNFSQILPDPSKP | 0.00 | 0.25 | 0.00 | 0.35 |
| QVKQIYKTPPIK**D**FGGFNFSQILPDPSKPS | 0.00 | 0.26 | 0.00 | 0.45 |
| VKQIYKTPPIK**D**FGGFNFSQILPDPSKPSK | 0.14 | 0.49 | 0.00 | 0.50 |
| KQIYKTPPIK**D**FGGFNFSQILPDPSKPSKR | 0.10 | 0.42 | 0.00 | 0.65 |
| QIYKTPPIK**D**FGGFNFSQILPDPSKPSKRS | 0.00 | 0.23 | 0.00 | 0.40 |
| IYKTPPIK**D**FGGFNFSQILPDPSKPSKRSF | 0.00 | 0.04 | 0.00 | 0.05 |
| YKTPPIK**D**FGGFNFSQILPDPSKPSKRSFI | 0.00 | 0.04 | 0.00 | 0.00 |
| KTPPIK**D**FGGFNFSQILPDPSKPSKRSFIE | 0.00 | 0.19 | 0.00 | 0.40 |
| TPPIK**D**FGGFNFSQILPDPSKPSKRSFIED | 0.00 | 0.23 | 0.00 | 0.15 |
| PPIK**D**FGGFNFSQILPDPSKPSKRSFIEDL | 0.00 | 0.04 | 0.00 | 0.00 |
| PIK**D**FGGFNFSQILPDPSKPSKRSFIEDLL | 0.02 | 0.04 | 0.00 | 0.00 |
| IK**D**FGGFNFSQILPDPSKPSKRSFIEDLLF | 0.00 | 0.13 | 0.00 | 0.00 |
| K**D**FGGFNFSQILPDPSKPSKRSFIEDLLFN | 0.05 | 0.15 | 0.00 | 0.05 |
| **D**FGGFNFSQILPDPSKPSKRSFIEDLLFNK | 0.00 | 0.51 | 0.00 | 0.35 |
